# The RLR/NLR expression and pro-inflammatory activity of tissue mast cells are regulated by cathelicidin LL-37 and defensin hBD-2

**DOI:** 10.1038/s41598-018-30289-w

**Published:** 2018-08-06

**Authors:** Justyna Agier, Sylwia Różalska, Magdalena Wiktorska, Paulina Żelechowska, Joanna Pastwińska, Ewa Brzezińska-Błaszczyk

**Affiliations:** 1 Department of Experimental Immunology, Faculty of Health Sciences, Medical University of Lodz, Lodz, Poland; 2 Department of Industrial Microbiology and Biotechnology, Faculty of Biology and Environmental Protection, University of Lodz, Lodz, Poland; 3 Department of Molecular Cell Mechanisms, Faculty of Health Sciences, Medical University of Lodz, Lodz, Poland; 4 Laboratory of Cellular Immunology, Institute of Medical Biology, Polish Academy of Sciences, Lodz, Poland

## Abstract

Considering the significance of mast cells (MCs) in the course of various physiological and pathological processes, and the pivotal role of endogenous molecules, i.e., cathelicidins and defensins as multifunctional modulators, the study examines the constitutive and cathelicidin LL-37/defensin hBD-2-induced expression of certain NLRs and RLRs, i.e., NOD1, NOD2, and RIG-I, in fully-mature tissue MCs, and the impact of LL-37 and hBD-2 on MC pro-inflammatory activity. All experiments were carried out *in vitro* on freshly-isolated peritoneal (P)MCs. qRT-PCR, western blotting, flow cytometry, and confocal microscopy were used to evaluate both constitutive and LL-37/hBD-2-induced expression of NOD1, NOD2, and RIG-I receptors. ROS was determined using H_2_DCFDA, and Boyden microchamber assay was used to define the migratory response. Standard techniques assessed histamine, cysLT, and chemokine generation. PMCs express NOD1, NOD2, and RIG-I constitutively. LL-37 and hBD-2 enhance the expression and induce translocation of the studied receptors and directly activate the pro-inflammatory and migratory responses of PMCs. Observations demonstrate that LL-37 and hBD-2 might augment MC capability and sensitivity to NLR and RLR ligands and strengthen the role of MCs in inflammation.

## Introduction

Host defense peptides (HDPs) are evolutionarily-conserved biologically-active molecules synthesized as the first line of defense by a variety of organisms. Due to their biochemical features, HDPs have commonly been referred to as cationic/amphipathic defense peptides. There is a remarkable diversity of HDPs across species, but cathelicidins and defensins constitute the major representatives of amphipathic peptides in vertebrates. They are synthesized in the form of abundant inactive precursors mainly by keratinocytes, epithelial cells and circulating phagocytic cells. It is assumed that cathelicidins and defensins are produced constitutively, but a significant majority are synthesized in response to the presence of pathogens or their products^1,2^. Cathelicidins and defensins have multidimensional properties allowing them to act directly against bacterial^3^, viral^4^ and fungal^5^ invasion but new research has cast light on alternative functionalities, including immunomodulatory activities. They can serve as chemokines, and stimulate the production and release of various immunoregulatory mediators by inflammatory cells; therefore, they are recognized as potent agents in inflammatory processes. Furthermore, cathelicidins and defensins can modulate adaptive immunity^6,7^. Besides their antimicrobial and modulatory activities, they also possess anticancer properties^8^.

Mast cells (MCs) are long-lived resident connective tissue cells distributed throughout the body. They are mainly numerous beneath the subepithelial layers of the skin, in the respiratory system, in the gastrointestinal and genitourinary tracts, and adjacent to blood vessels and nerves^9^. The cytoplasm of MCs contains 50–200 large granules that store abundant numbers of biologically-active granule-associated preformed mediators such as histamine, proteases, proteoglycans, and metalloproteinases. MCs are also a rich source of *de novo* generated arachidonic acid metabolites, e.g., leukotrienes (LTs), prostaglandins (PGs), thromboxanes (TXs), as well as many newly-synthesized cytokines and chemokines. MCs hence constitute the chief sentinels of the immune system, with a multiplicity of functions in the maintenance of a range of physiological features. They are considered crucial for the regulation of body homeostasis by acting on wound healing, angiogenesis, and vascular permeability, as well as taking part in the homeostasis of tissues and organs undergoing continuous growth and remodeling. They also strongly influence both innate and acquired immune responses. Likewise, MCs have been implicated in a variety of pathological conditions, including allergic processes and carcinogenesis. Notably, MCs manage both acute and chronic inflammation and play a prominent role in inflammatory diseases^10–14^. Beyond the above, MCs act as efficient effector cells in microbial elimination and play an essential role in orchestrating inflammatory response during infection^15,16^. Furthermore, MCs can phagocytose and subsequently kill bacteria, *via* oxidative and non-oxidative systems^17,18^ and contribute to host defense by forming extracellular traps (MCETs), which can entrap and eliminate various microbes^19^. Also, they can release not only active mediators but cathelicidins^20^. Following phagocytosis, MCs have the capability of processing bacterial antigens for presentation through class I and II MHC molecules, which leads to the development of adaptive antimicrobial immunity^18,21^.

As MCs play such a significant role in the course of various physiological and pathological processes and considering the pivotal role of endogenous molecules as multifunctional modulators, we chose to examine the relationship between cathelicidins or defensins and MCs. These findings paved the way for research on the impact of HDPs on pattern recognition receptors (PRRs), which bind not only to pathogen-associated molecular patterns (PAMPs) but importantly, also to the endogenous molecules termed damage-associated molecular patterns (DAMPs) released from stressed or dying cells. We have been found that LL-37 affects Toll-like receptor (TLRs) expression, enhancing TLR2, TLR4, and TLR9 on the MC surface, and TLR3, TLR5, and TLR7 in the cell interior^22^. Previously, Yoshioka *et al*.^23^ had observed a relationship between receptor level and LL-37 in the case of TLR4.

Little, if any, information exists regarding whether *in vivo* differentiated mature tissue MCs isolated from the rat peritoneal cavity (PMCs) express nucleotide-binding oligomerization domain (NOD)-like receptors (NLRs) and retinoic acid-inducible gene I (RIG-I)-like receptors (RLRs), as well as the impact of LL-37 and defensin hBD-2 on this expression. Therefore, the purpose of the current study was to examine the constitutive and LL-37/hBD-2-induced expression of representative NLRs and RLRs, i.e., NOD1, NOD2, and RIG-I in PMCs. It also discusses the effects of LL-37 and hBD-2 on PMC pro-inflammatory activity.

## Materials and Methods

### Ethics statement

Animals used in this study were handled according to the European guideline (2010/63/EU) and the Act on the protection of animals used for scientific or educational purposes (Dz.U. 2015, poz. 266). The experimental protocols were approved by the Local Ethics Committee for Experiments on Animals in Lodz (the approval No. 20/ŁB740/2015). All efforts were made to minimize animal suffering. From the humanitarian aspect, female rats were selected due to the higher number of MCs in peritoneal cavity than males. All animals were treated with isoflurane-induced anesthesia before decapitation.

### Animals

The study was performed on female albino Wistar rats Crl:WI (Charles River Laboratories) weighing ~250 g, aged three to four months. The animals were obtained from the animal quarters of the Faculty of Biology and Environmental Protection of the University of Lodz. Standard storage conditions for animal breeding were provided: the animals were kept in metal cages, five rats in each, at room temperature. They were maintained under artificial lighting for a 12-hour light cycle. The animals were fed with Murigran granulated fodder for rodents and water *ad libitum*.

### Isolation of PMCs

Peritoneal cell suspensions were obtained from peritoneal cavities by lavage with 50 mL of 1% HBSS (GIBCO, Gaithersburg, MD, USA) supplemented with 0.015% sodium bicarbonate (GIBCO). After abdominal massage (90 sec) the cell suspension was removed from the peritoneal cavity. The peritoneal cell suspension was washed twice (150 *g*, 5 min, 20 °C) in complete (c)DMEM containing DMEM (Biowest, Kansas City, MO, USA), supplemented with 10% FCS (GIBCO), 10 μg/mL gentamicin (GIBCO) and 2 mM glutamine (GIBCO). Isotonic 72.5% Percoll (Sigma-Aldrich, St. Louis, MO, USA) density gradient centrifugation (190 *g*, 15 min, 20 °C) was used for PMC purification. Subsequently, isolated PMCs were centrifuged twice in cDMEM (150 *g*, 5 min, 20 °C). The isolation of PMCs lasted approximately 45–50 minutes. After being washed, PMCs were counted and resuspended in an appropriate volume of cDMEM (for quantitative RT-PCR, flow cytometry analysis, confocal microscopy technique, CCL2 and CCL3 release measurements, migration assay) or medium for rat PMCs, containing 137 mM NaCl (Sigma-Aldrich), 2.7 mM KCl (Sigma-Aldrich), 1 mM MgCl_2_ (Sigma-Aldrich), 1 mM CaCl_2_ (Sigma-Aldrich), 10 mM HEPES (Sigma-Aldrich), 5.6 mM glucose (Sigma-Aldrich), and 1 mg/mL BSA (Sigma-Aldrich) (for histamine release assay and cysLT synthesis measurement), to obtain PMC concentration of 1.5 × 10^6^ cells/mL. To acquire appropriate PMC density and number of samples in given type of experiment, proper number of animals was used. MCs were prepared with purity >98%, as determined by metachromatic staining with toluidine blue (Sigma-Aldrich). The viability of PMCs was over 98%, as determined by trypan blue (Sigma-Aldrich) exclusion assay. The results of treated samples were compared to the control from a given experiment.

### Quantitative RT-PCR

qRT-PCR was used to determine NOD1, NOD2, RIG-I mRNA levels. Purified PMCs suspended in cDMEM were stimulated with LL-37 (AnaSpec, Fremont, USA) or hBD-2 (PeptaNova GmbH, Sandhausen, DE) at a final concentration of 1 µg/mL for two hours at 37 °C in a humidified atmosphere with 5% CO_2_. For control, PMCs were incubated under the same conditions without hBD-2/LL-37. Total RNA was isolated from cells using TRI Reagent (Sigma Aldrich) and then reverse transcribed using a High Capacity cDNA Reverse Transcription Kit (Applied Biosystems, Foster City, USA). For qRT-PCR, TaqMan® probes dyed 6-carboxyfluorescein (rat Nod1 Rn01410158, rat Nod2 Rn01770864, rat Ddx58 Rn01439703, rat ACTB Rn00667869) (Applied Biosystems) and TaqMan® Gene Expression Master Mix (Applied Biosystems) were used. All reactions were performed with the use of the 7900 HT Fast Real-Time PCR System (Applied Biosystems). The amplification conditions were as follows: initial denaturation at 95 °C for 20 s, followed by 40 cycles of amplification: 95 °C for 3 s and 60 °C for 30 s. The RQ of testes samples was calculated by the SDS RQ Manager software, based on ΔΔCt method. The expression of receptor mRNAs was corrected by normalization based on the transcript level of the housekeeping gene *rat ACTB*. As the calibrator samples, unstimulated specimens were used.

### Western blotting

For determination of constitutive and LL-37/hBD-2-induced expression of NOD1, NOD2, and RIG-I immunoblotting was used. Purified PMCs were incubated with LL-37 or hBD-2 at a final concentration of 1 µg/mL or medium alone (non-stimulated cells; NS) for one or three hours. After incubation, the medium was discarded and PMCs were washed with ice-cold PBS. Cell pellets were lysed with RIPA buffer (Sigma-Aldrich) on ice for 20 min and centrifuged. Protein concentrations were measured using the Pierce BCA Protein Assay (Thermo Fisher Scientific, Waltham, MA, USA). Proteins (40 µg total) were separated on 11% SDS-PAGE gels and then transferred (two hours, 200 mA, 4 °C) onto nitrocellulose membranes (Bio-Rad, Hercules, CA, USA). The membranes were blocked with 5% BSA (Sigma Aldrich) in PBS for one hour at room temperature. Next, the membranes were incubated with primary antibodies overnight at 4 °C. The final dilutions for primary antibodies were as follow: rabbit polyclonal anti-NOD1 (Santa Cruz Biotechnology Inc., Dallas, TX, USA) (1:200), rabbit polyclonal anti-NOD2 (Santa Cruz Biotechnology Inc.) (1:200), goat polyclonal anti-RIG-I (Santa Cruz Biotechnology Inc.) (1:200), and rabbit polyclonal anti-ACTB (Abcam, Cambridge, UK) (1:200). Following three washes, the membranes were incubated with horseradish peroxidase-conjugated secondary antibodies (Dako, Carpinteria, CA, USA) diluted at 1:1000 (for NOD1, NOD2, ACTB) or alkaline phosphatase-conjugated secondary antibodies (Santa Cruz Biotechnology, Inc., Dallas, USA) diluted at 1:1000 (for RIG-I) in PBS with 0.05% TWEEN20 (Sigma-Aldrich) containing 1% BSA for two hours at room temperature. Immunoblots were visualized using Pierce ECL Western Blotting Substrate (Thermo Fisher Scientific) and Kodak BioMax Light Film (Eastman Kodak, Rochester, NY, USA) or BCIP/NBT (Santa Cruz Biotechnology, Inc.). Spectra Multicolor broad range protein ladder (10 kD to 260 kD) was from Thermo Scientific. The developed images were scanned and protein band intensity was quantified by ImageJ Software.

### Cell preparation for flow cytometric and confocal microscopy analysis

Constitutive and LL-37/hBD-2-induced NOD1, NOD2 and RIG-I expression were determined using flow cytometry and confocal microscopy. Constitutive expression of NLRs and RIG-I was assessed in native PMCs (non-stimulated cells). Induced receptor expression was estimated in PMCs incubated with LL-37 or hBD-2, at a final concentration of 1 µg/mL, for one or three hours at 37 °C in a humidified atmosphere with 5% CO_2_. Following this, the PMCs were fixed with CellFIX (BD Bioscience, San Jose, USA) solution for 15 minutes at 4 °C and washed twice with 1 x PBS (Cayman Chemical, Ann Arbor, USA). To determine the intracellular localization of receptors, the PMCs were permeabilized with 0.1% saponin (Sigma-Aldrich) for 30 minutes at room temperature. Next, PMCs were resuspended in 1 x PBS and stained for one hour with rabbit anti-NOD1, rabbit anti-NOD2, and goat anti-RIG-I antibodies (Santa Cruz Biotechnology, Inc.) (dilution 1:100). For control, PMCs were stained with goat or rabbit IgG isotype control (R&D Systems, Minneapolis, USA) with irrelevant specificity. Primary antibody was not added to the sample to certify non-specific binding of the secondary antibody. Cells were then washed with 1 x PBS and incubated with Alexa Fluor 488® rabbit anti-goat IgG or Alexa Fluor 488® goat anti-rabbit IgG (Jackson ImmunoResearch Laboratories, Inc., West Grove, USA) (dilution 1:100) in 1 x PBS for one hour in the dark. Following this, the cells were washed twice and finally resuspended in 1 x PBS before receptor assessment. After each period of incubation, PMC viability was examined using the trypan blue exclusion test.

To indicate ROS generation by confocal microscopy, PMCs were incubated with LL-37 or hBD-2 at 1, 10, 20 µg/mL or medium alone for 30 minutes in at 37 °C a humidified atmosphere with 5% CO_2_. Indicator for ROS, H_2_DCFDA (Sigma-Aldrich) was used at a concentration of 2 μM for 10 minutes. Following this, the cells were washed and resuspended in 1 x PBS.

### Flow cytometry

Ten thousand events in each sample were analyzed using a FACSCalibur flow cytometer with CellQuest software (BD Biosciences). LL-37/hBD-2-dependent PMC NOD1, NOD2, and RIG-I expression were presented as a percentage of NOD1/NOD2/RIG-I MFI (mean fluorescence intensity) measured in native PMCs (referred to as 100%).

### Confocal microscopy

The samples were mounted on microscope slides, and images were captured using an LSM 510 Meta confocal laser scanning microscope (Zeiss, Oberkochen, Germany) combined with an Axi1overt 200 M (Zeiss) inverted microscope equipped with a Plan-Neofluar objective (40x/0.6). All settings were held constant throughout the experiments except for gain factor, which was adjusted for each receptor. The fluorescence was recorded using the argon laser (488 nm) and a BP filter set (505 nm). The same laser line was used for Nomarski DIC. All signals obtained from confocal microscopy were validated with profile view image analysis and the diagrams presenting intensity values placed beside each microphotograph. The mean fluorescence intensity (expressed in arbitrary units AU) was calculated for each of the samples. The calculations were performed for at least 40 different points randomly selected in compartments with receptor expression.

### ELISA

For cytokine generation measurements, purified PMCs suspended in cDMEM were incubated with LL-37 or hBD-2 at final concentrations of 1, 5, 10, 20 or 40 μg/mL anti-IgE (AbD Serotec, Oxford, UK) at a final concentration of 5 μg/mL (positive control) or buffer alone (spontaneous chemokine generation) in a humidified atmosphere with 5% CO_2_ for three hours at 37 °C. The supernatants were collected by centrifugation. CCL2 and CCL3 concentrations in supernatants were evaluated by ELISA kits (Cloud-Clone Corp., Katy, USA, Wuhan Fine Biotech Co., Ltd, Wuhan, China) according to the manufacturer’s instructions. The sensitivity of these tests was <15 pg/mL and <59 pg/mL, respectively.

For cysLT synthesis analysis, purified PMCs suspended in rat PMC medium were incubated with LL-37 or hBD-2 at final concentrations of 1, 5, 10, 20 or 40 μg/mL, calcium ionophore A23187 (Sigma-Aldrich) at a final concentration of 5 μg/mL (positive control) or buffer alone (spontaneous release) in a water bath for one hour at 37 °C with constant stirring. The supernatants were collected by centrifugation. The concentration of cysLTs in supernatants was evaluated by ELISA kit (Cayman Chemical) according to the manufacturer’s instructions. The sensitivity of the assay was <13 pg/mL.

### Histamine-release assay

Purified PMCs suspended in medium for rat PMCs were incubated with LL-37 or hBD-2 at final concentrations of 1, 5, 10, 20, and 40 µg/mL, or compound 48/80 (Sigma-Aldrich), a commonly-known potent MC degranulation factor, at a final concentration of 5 µg/mL (positive control) or buffer alone (spontaneous histamine release) in a water bath for 30 minutes at 37 °C with constant stirring. For time-course experiments, PMCs were incubated with LL-37/hBD-2 at a final concentration of 40 μg/mL for 0, 1, 3, 5, 10, or 30 min. After incubation, the reaction was stopped by adding 1.9 mL of cold medium. Next, the cell suspension was centrifuged, and the supernatants were demounted into other tubes. A total of 2 mL distilled water was added to each tube with the cell pellets. Histamine content was determined in both cell pellets (residual histamine) and supernatants (released histamine) by the spectrofluorometric method, as previously described^24^. Histamine release was expressed as a percentage of the total cellular content of the amine.

### Migration assay

The PMC migratory response to LL-37 and hBD-2 was examined *in vitro* using Boyden microchamber assay (Neuro Probe, Gaithersburg, USA) in a 48-well chemotaxis chamber (Neuro Probe). Thirty microliters of LL-37 or hBD-2, at final concentrations of 1, 5, 10, 20, and 40 µg/mL, or tumor necrosis factor (TNF) (R&D Systems) at a final concentration of 0.05 pg/mL (positive control), or buffer alone (control spontaneous migration) was placed into the lower compartments of microchamber. The lower compartments were covered with a polycarbonate 8-μm-pore-size membrane, and 50 μL of the cell suspensions were applied to the upper compartments. Subsequently, the chemotaxis chamber was incubated for three hours in a humidified atmosphere with 5% CO_2_ at 37 °C. After the incubation period, PMCs adherent to the upper surface of the membrane was removed by scraping with a rubber blade. Migrating cells adherent to the lower surface of the membrane were fixed in 99.8% ethanol, stained for 10 min with hematoxylin, cleared in distilled water, and mounted on a microscope slide. PMC migration was quantified by counting the number of cells that had traversed the membrane and were attached to the bottom surface of the filter. Ten high-power fields (HPF) were calculated in each assay (x250). Spontaneous migration served as a control and was referred to as 100%. The results were presented as a percentage of control migration.

### Statistical analysis

The statistical analysis of the experimental data was performed using Statistica 13 software (Statsoft Inc., USA). Data are presented as mean ± SD. Normality of distribution was tested with the Shapiro-Wilk test. All comparisons between groups were carried out by using Student’s *t*-test for small groups or one-way ANOVA. Differences were considered significant at *P* < 0.05 and are labeled with an asterisk (*) on each graph.

## Results

### Effect of LL-37 and hBD-2 on NOD1, NOD2, and RIG-I expression

**Figure 1:**
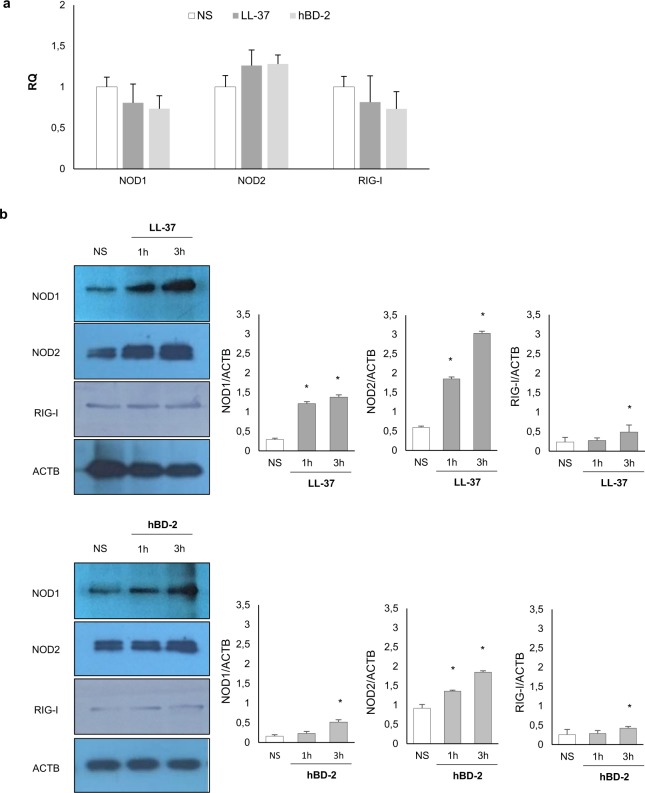
Constitutive and LL-37/hBD-2-induced NOD1, NOD2, and RIG-I mRNA and protein expression in PMCs. PMCs were incubated with medium alone (native, non-stimulated cells; NS), LL-37 or hBD-2 at a final concentration of 1 µg/mL. Receptor expression was assessed by (**a**) qRT-PCR and (**b**) WB. ACTB was used as control. Data represents the mean ± SD of three independent experiments performed with duplicate samples (each experiment was performed on 4 animals/PMC isolates). Differences were considered significant at *P* < 0.05 and are labeled with an asterisk (*) on each graph (Student’s *t*-test). One representative blot of three independent experiments performed with duplicate samples is shown. The cropped blots are used in the figure, and the full-length blots are presented in Supplementary Fig. S1.

The qRT-PCR and western blotting were used to determine the expression of NOD1, NOD2, and RIG-I mRNAs and proteins by native PMCs, and those stimulated with LL-37 or hBD-2. As demonstrated in Fig. 1a, PMCs express mRNA for all studied receptors constitutively. LL-37 and hBD-2 did not affect NOD1, NOD2 or RIG-I mRNA expression. As shown in Fig. 1b, NOD1, NOD2, and RIG-I were significantly upregulated upon incubation with LL-37 or hBD-2 after three hours incubation.

**Figure 2:**
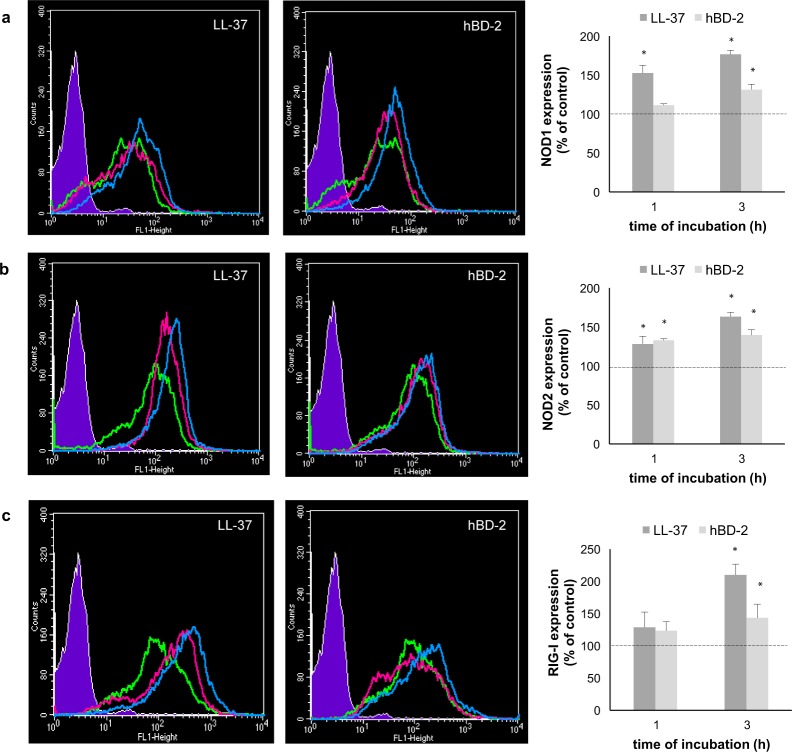
Effect of LL-37 and hBD-2 stimulation on (**a**) NOD1, (**b**) NOD2, (**c**) RIG-I expression in PMCs. PMCs were incubated with medium alone (non-stimulated cells; NS), LL-37 or hBD-2 at a final concentration of 1 µg/mL. Left panel: Representative flow cytometry histogram showing NOD1, NOD2, and RIG-I expression. Shaded tracings – isotype control, open tracings – receptor expression in native cells (green) and after LL-37/hBD-2 stimulation for one h (violet) and three h (blue). Right panel: Constitutive receptor expression served as a control and was referred to as 100%. The results are presented as percentage of constitutive receptor expression. Data represents the mean ± SD of three independent experiments performed with duplicate samples (each experiment was performed on 4 animals/PMC isolates). Differences were considered significant at *P* < 0.05 and are labeled with an asterisk (*) on each graph (Student’s *t*-test).

Next, we investigated whether PMCs express NOD1, NOD2, and RIG-I proteins and whether LL-37 and hBD-2 have an impact on their baseline levels. Receptor expression was evaluated on native PMCs, as well as on PMCs exposed to LL-37 or hBD-2 at a concentration of 1 µg/mL for one or three hours. The flow cytometry analysis revealed that native PMCs expressed intracellular NOD1, NOD2 and RIG-I receptors (Fig. 2a–c). It was found that the baseline level of NOD1 expression was significantly up-regulated (*P* < 0.05) following one and three-hour incubation with LL-37, reaching 152.46 ± 10.18% and 176.55 ± 5.35% of control NOD1 expression in native PMCs, respectively (Fig. 2a). The three-hour incubation of PMCs with hBD-2 resulted in a statistically significant (*P* < 0.05) increase in NOD1 level compared with the control unstimulated PMCs (131.23 ± 6.9% of control NOD1 expression). As shown in Fig. 2b, both LL-37 and hBD-2 enhanced NOD2 protein level, and the intensity of the signals were the highest after three hours of stimulation. Three-hour PMC stimulation with LL-37 and hBD-2 resulted in increased RIG-I expression compared to non-stimulated cells (210.1 ± 16.53% and 143.47 ± 21.09% of control RIG-I expression) (Fig. 2c).

**Figure 3:**
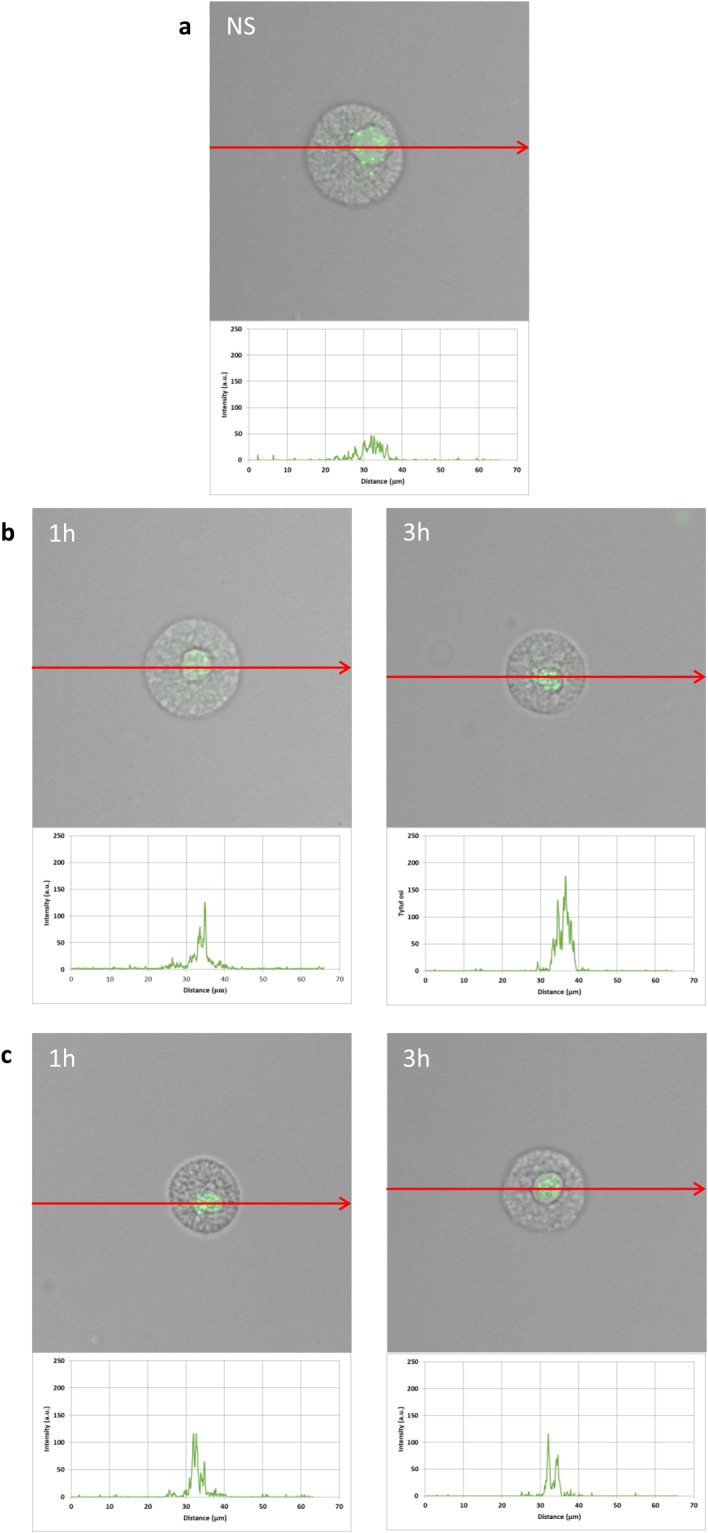
Effect of LL-37 and hBD-2 stimulation on NOD1 expression in PMCs. PMCs were incubated with medium alone (non-stimulated cells; NS), LL-37 or hBD-2 at a final concentration of 1 µg/mL. Representative images showing NOD1 cellular localization in permeabilized (**a**) native, (**b**) LL-37 stimulated, (**c**) hBD-2 stimulated PMCs analyzed by confocal microscopy. Single confocal sections (midsection of cells) reveal the presence of NOD1. One representative of three independent experiments performed with duplicate samples (each experiment was performed on 4 animals/PMC isolates) is shown. Fluorescence intensity diagrams showing the distribution of fluorescence in cells were mounted.

To assess the cellular distribution of NOD1, NOD2, and RIG-I, confocal microscopy was used. PMCs were stained for intracellular expression of all studied receptors. Isotype controls and controls for non-specific binding of the secondary antibody confirmed the specificity of antibodies (data not shown). The confocal microscopy and image analysis confirmed the presence of NOD1 in native PMCs (Fig. 3a). The signals were strongly associated with the cytoplasm located near the cell nucleus. PMC stimulation with LL-37 resulted in an increase of NOD1 expression, which was documented by intensity diagrams beside each microphotograph (Fig. 3b). After one hour of incubation with hBD-2, the intensity of the signals was also stronger in comparison to controls, i.e., 52.1 ± 22.0 fluorescence intensity arbitrary units (AU) compared to 26.7 ± 8.9 AU, and this level was maintained until three hours of incubation (Fig. 3c). The above observations are in good agreement with flow cytometric analysis.

**Figure 4:**
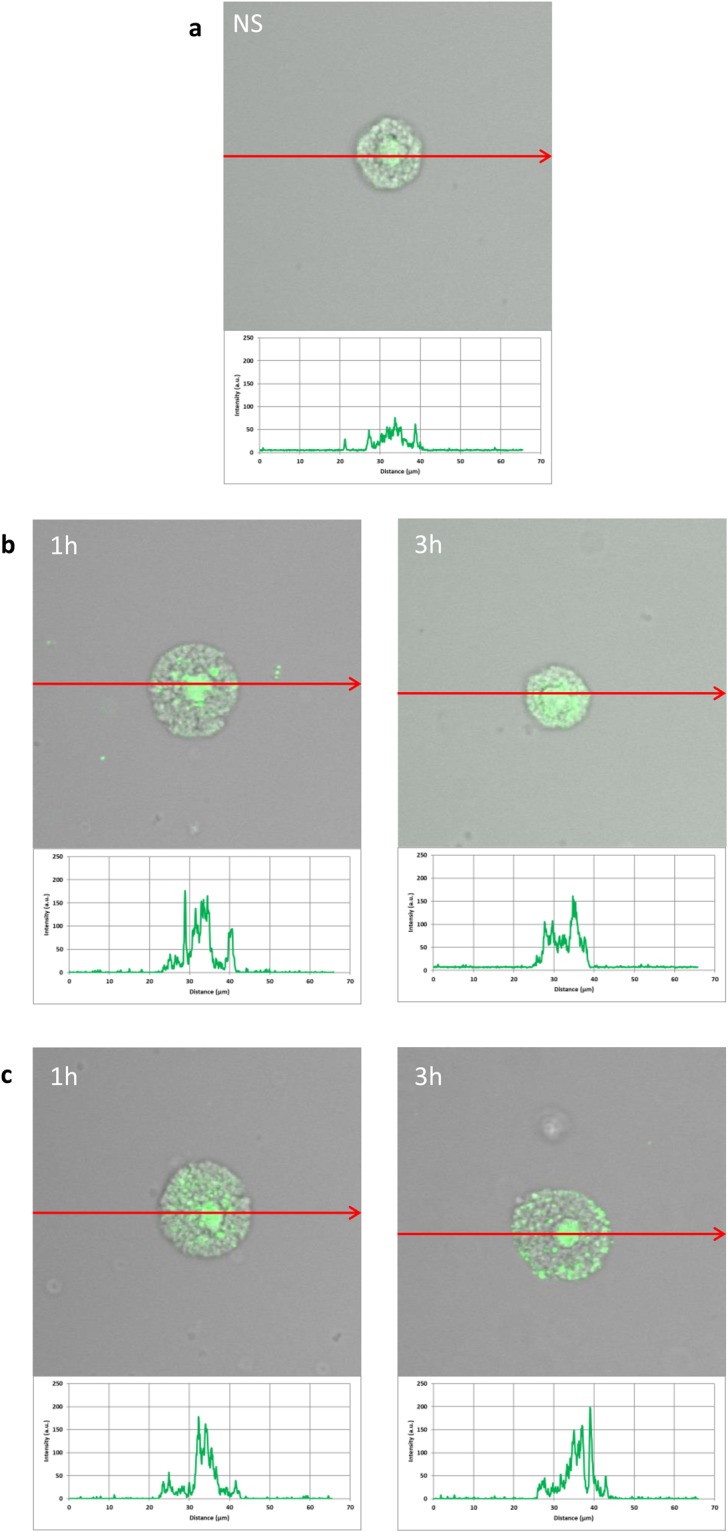
Effect of LL-37 and hBD-2 stimulation on NOD2 expression in PMCs. PMCs were incubated with medium alone (non-stimulated cells; NS), LL-37 or hBD-2 at a final concentration of 1 µg/mL. Representative images showing NOD2 cellular localization in permeabilized (**a**) native, (**b**) LL-37 stimulated, (**c**) hBD-2 stimulated PMCs analyzed by confocal microscopy. Single confocal sections (midsection of cells) reveal the presence of NOD2. One representative of three independent experiments performed with duplicate samples (each experiment was performed on 4 animals/PMC isolates) is shown. Fluorescence intensity diagrams showing the distribution of fluorescence in cells were mounted.

**Figure 5:**
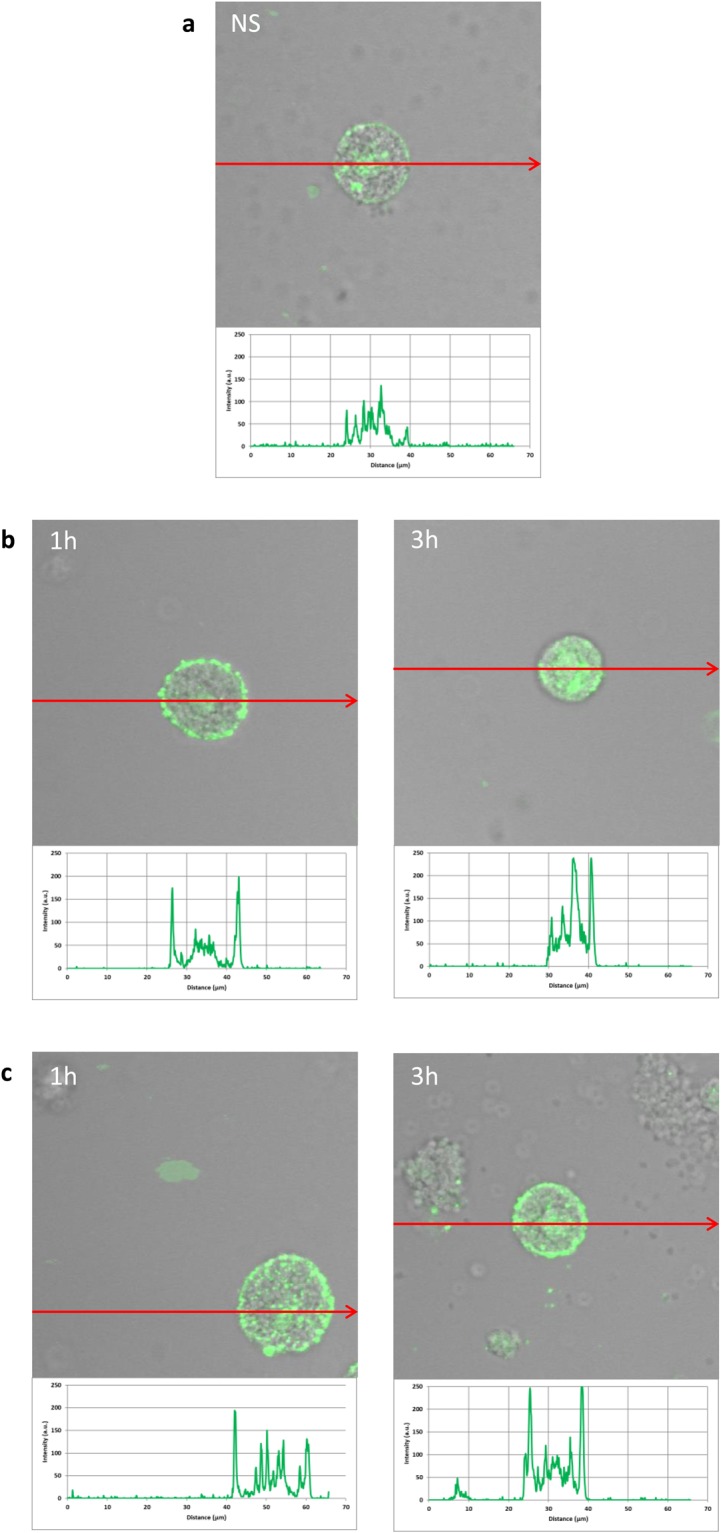
Effect of LL-37 and hBD-2 stimulation on RIG-I expression in PMCs. PMCs were incubated with medium alone (non-stimulated cells; NS), LL-37 or hBD-2 at a final concentration of 1 µg/mL. Representative images showing RIG-I cellular localization in permeabilized (**a**) native, (**b**) stimulated LL-37, (**c**) stimulated hBD-2 PMCs analyzed by confocal microscopy. Single confocal sections (midsection of cells) reveal presence of RIG-I. One representative of three independent experiments performed with duplicate samples (each experiment was performed on 4 animals/PMC isolates) is shown. Fluorescence intensity diagrams showing the distribution of fluorescence in cells were mounted.

The expression of NOD2 by PMCs is shown in Fig. 4. Immunocytochemical staining indicated that fluorescence is predominantly associated with the cytosol and endoplasmic reticulum (Fig. 4a). Additionally, NOD2 expression (39.7 ± 12.8 AU) is greater than NOD1 (26.7 ± 8.9 AU). PMC stimulation with LL-37 resulted in a significant enrichment in the signals, not only in the intracellular regions but also beneath the cell surface, as seen in intensity diagrams beside each microphotograph (Fig. 4b). Likewise, incubation with hBD-2 caused an increase in NOD2 intracellular expression compared with non-stimulated cells (Fig. 4c), with a significant fluorescence signal being associated with the endoplasmic reticulum after one hour of stimulation (94.9 ± 37.4 AU). In turn, incubation with hBD-2 for an extended time caused an increase in NOD2 expression at the cell surface in about 25% compared with non-stimulated cells. As demonstrated in Fig. 5a, RIG-I is located not only in the intracellular space but also below the cell membrane of native cells. In the interior of non-stimulated permeabilized cells, a similar fluorescence signal was found to be associated with the cytoplasm and outer membrane. PMC treatment with LL-37 caused an enhancement of RIG-I near the cell surface after one hour of incubation, which was confirmed by intensity diagram analysis (Fig. 5b). The intensity of the signals was higher after three hours (115.6 ± 25.4 AU) in comparison to non-stimulated cells (45.4 ± 15.2 AU), but it appeared that the significant receptor pool is stored not on the surface, but intracellularly. hBD-2 increased expression of RIG-I after one and three-hour exposure times (Fig. 5c).

### Effect of LL-37 and hBD-2 on PMC pro-inflammatory response

**Figure 6:**
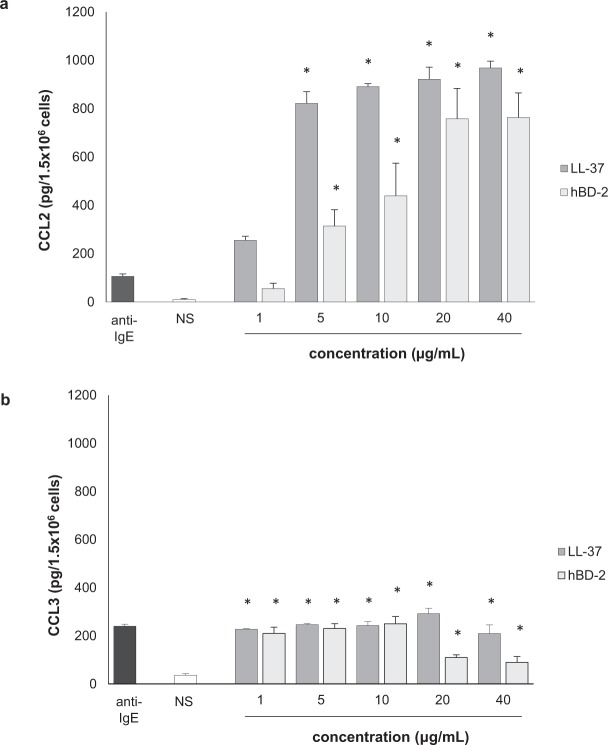
Effect of LL-37 on (**a**) CCL2 and (**b**) CCL3 synthesis in PMCs. For CCL2 and CCL3 measurement, PMCs were incubated with different concentrations of LL-37 and hBD-2, anti-IgE at 5 μg/mL (positive control) or medium alone (NS). Data represents the mean ± SD of four independent experiments (each experiment was performed on 2 animals/PMC isolates). Comparisons between groups were carried out by using Student’s *t*-test for small groups. Differences were considered significant at *P* < 0.05 and are labeled with an asterisk (*) on each graph (Student’s *t*-test).

To confirm whether LL-37 and hBD-2 could provoke a pro-inflammatory response by MCs, we first evaluated the abilities of these peptides to induce chemokine generation and release. To this end, PMCs were stimulated with LL-37 or hBD-2 at concentrations of 1, 5, 10, 20, and 40 µg/mL for three hours, using the medium alone as a negative control or anti-IgE as a positive control. After incubation, the levels of CCL2 and CCL3 in supernatants were determined by specific ELISA kits. The results of these experiments are shown in Fig. 6. It was found that both LL-37 and hBD-2 peptides induced CCL2 release, with LL-37 being more potent than hBD-2 (Fig. 6a). Of the various concentrations of LL-37 or hBD-2, the greatest chemokine secretion was observed at 40 µg/mL, rising to 967.75 ± 28.69 pg/1.5 × 10^6^ cells and 762.54 ± 102.38 pg/1.5 × 10^6^ cells, respectively. Statistically significant differences were determined between means by one-way ANOVA (*P* = 0.018). Similarly, both peptides induced a considerable release of CCL3, comparable to anti-IgE-induced chemokine release (Fig. 6b). When treated with 20 µg/mL LL-37, chemokine secretion by PMCs peaked at 292.3 ± 22.48 pg/1.5 × 10^6^ cells, before decreasing. hBD-2 at concentrations of 1, 5, and 10 µg/mL was also found to be capable of triggering significant CCL3 production by PMCs compared to controls (*P* = 0.021, one-way ANOVA).

**Figure 7:**
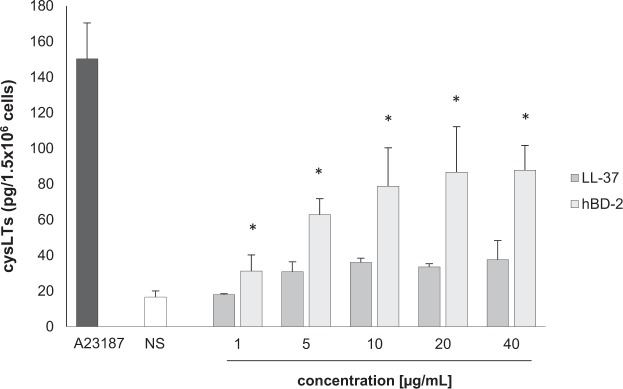
Effect of LL-37 and hBD-2 on PMC cysLT synthesis and release. PMCs were incubated with different concentrations of LL-37 or hBD-2, calcium ionophore A23187 at 5 μg/mL (positive control) or medium alone (NS). Data represents the mean ± SD of three independent experiments performed with duplicate samples (each experiment was performed on 2 animals/PMC isolates). Differences were considered significant at *P* < 0.05 and are labeled with an asterisk (*) on each graph (Student’s *t*-test).

The next stage examined cysLT generation and release. PMCs were incubated for one hour with LL-37 and hBD-2 at concentrations of 1, 5, 10, 20, and 40 µg/mL; a negative control was medium alone while a positive control was calcium ionophore A23187. As shown in Fig. 7, LL-37 did not stimulate cysLT production and release by PMCs. However, the cysLT release of 31.19 ± 9.01 pg/1.5 × 10^6^ cells to 87.80 ± 13.84 pg/1.5 × 10^6^ cells was observed following exposure to 1 to 40 µg/mL hBD-2, and up to 150.32 ± 20.13 pg/1.5 × 10^6^ cells was seen when treated with calcium ionophore A23187.

**Figure 8:**
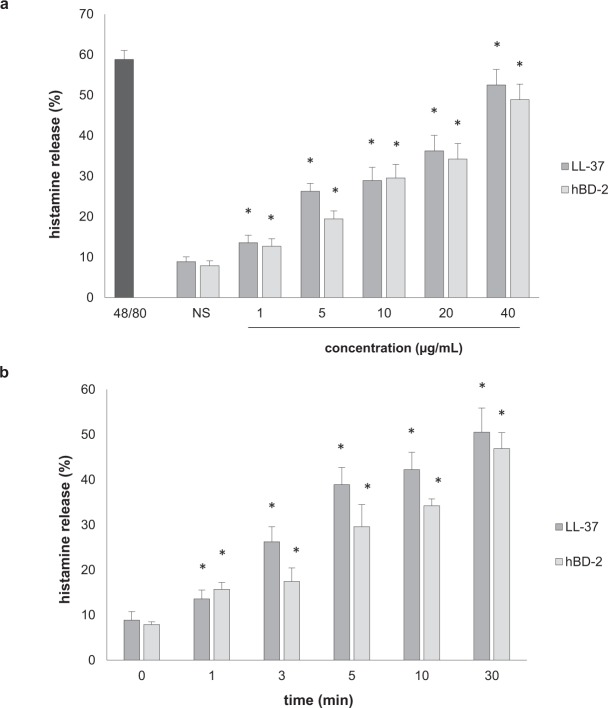
Effect of LL-37 and hBD-2 on PMC histamine release. (**a**) PMCs incubated with different concentrations of LL-37 or hBD-2, or compound 48/80 at 5 μg/mL (positive control) or medium alone (NS) for 30 min. (**b**) MCs were stimulated with LL-37 or hBD-2 at 40 μg/mL at the indicated time periods. Data represents the mean ± SD of three independent experiments performed with duplicate samples (each experiment was performed on 2 animals/PMC isolates). Differences were considered significant at *P* < 0.05 and are labeled with an asterisk (*) on each graph (Student’s *t*-test).

LL-37 and hBD-2 also activated PMCs to dose-dependent degranulation at all tested concentrations, as assessed by histamine secretion (Fig. 8). PMCs challenged with LL-37 and hBD-2 at 40 µg/mL released up to 52.0 ± 7.0% and 47.0 ± 9.5% of histamine, respectively (Fig. 8a). For comparison, a potent degranulation inducer, i.e., compound 48/80, induced PMC histamine secretion up to 58.0 ± 6.0% upon 30 minutes of incubation. Time-course experiments revealed statistically significant (*P* = 0.037, one-way ANOVA) histamine release within five minutes of incubation with LL-37 and hBD-2 (Fig. 8b).

**Figure 9:**
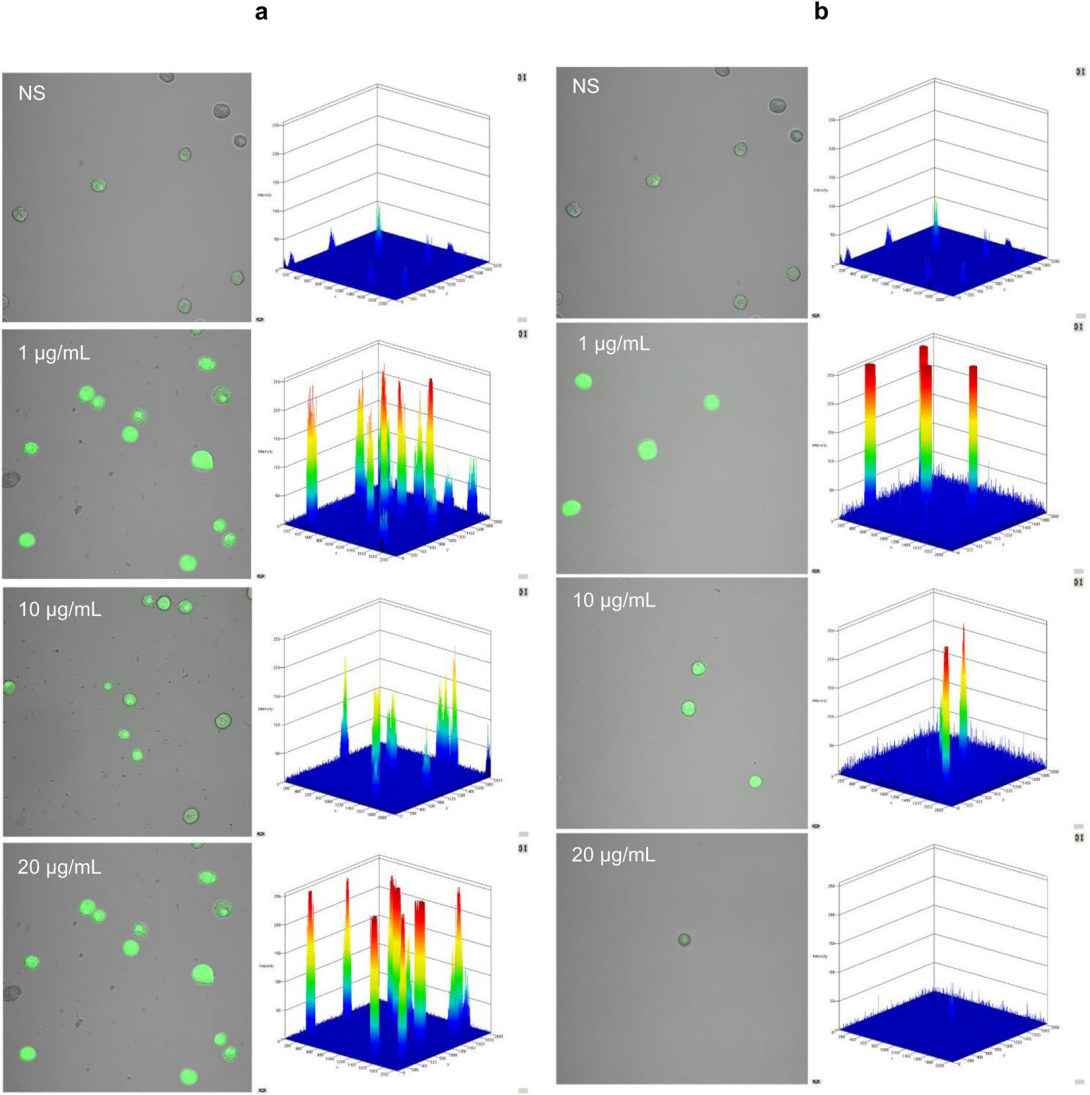
Effect of LL-37 (A) and hBD-2 (B) on ROS production by PMCs. To evaluate ROS generation, PMCs were incubated with 1, 5, 10 µg/mL (**a**) LL-37 or (**b**) hBD-2 or medium alone (NS). Indicator for ROS, H2DCFDA, was used at a concentration of 2 μM for 10 min. One representative of three independent experiments performed with duplicate samples (each experiment was performed on 2 animals/PMC isolates) is shown. Fluorescence intensity diagrams showing the distribution of fluorescence in cells were mounted.

Finally, confocal microscopy was used to study the influence of 1, 10 and 20 µg/mL LL-37 and hBD-2 on ROS generation (Fig. 9). At the lowest concentration, LL-37 treatment induced fourfold greater ROS production compared to controls, and hBD-2 treatment induced fivefold greater production. At 10 µg/mL, both agents increased the basal level of ROS by approximately threefold. Interestingly, at 20 µg/mL, although the level of ROS remained around fourfold higher following LL-37 treatment, the difference was negligible in the case of hBD-2.

### Effect of LL-37 and hBD-2 on PMC migration

**Figure 10:**
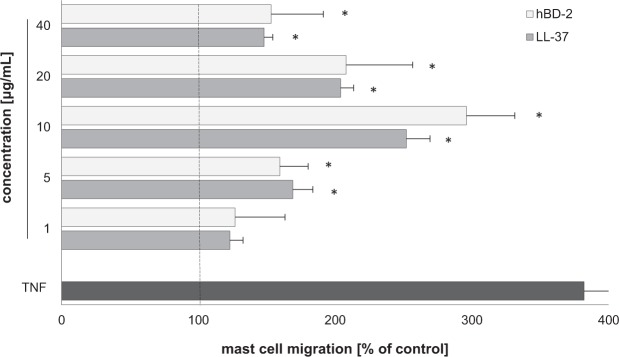
Effect of LL-37 and hBD-2 on PMC migratory response. PMCs were incubated with different concentrations of LL-37, TNF at 0.05 pg/mL (positive control) or medium alone (spontaneous mast cell migration) in a Boyden microchamber. Ten HPF were counted in each assay (×250), and each sample was tested in duplicate. Spontaneous migration served as a control and was referred to as 100%. Results are expressed as a percentage of spontaneous migration. Data represents the mean ± SD of three independent experiments performed with duplicate samples (each experiment was performed on 2 animals/PMC isolates). Differences were considered significant at *P* < 0.05 and are labeled with an asterisk (*) on each graph (Student’s *t*-test).

PMCs were incubated with LL-37 and hBD-2 for three hours in a Boyden microchamber, at a wide range of concentrations, to determine their capability to induce PMC migration. These results were compared with the migration occurring to TNF, a well-known MC chemotactic factor. Results indicate that both LL-37 and hBD-2 at concentrations from 1 to 40 µg/mL strongly induced PMC migration, as compared to spontaneous migration (Fig. 10). It was found that 10 µg/mL LL-37 induced maximal PMC migration (252.74 ± 17.4% of control migration) and that the PMCs were observed to migrate toward hBD-2 at this concentration (296 ± 35.4% of control migration). At the same experimental conditions migration of PMCs in response to TNF was up to 382.5 ± 30.3% of control migration.

## Discussion

To detect pathogens, the mammalian immune system has evolved receptors identifying encountered non-mammalian molecular patterns. For example, NOD1 and NOD2 identify distinct motifs of peptidoglycan (PGN) (i.e., D-γ-glutamyl-meso-DAP and muramyl dipeptides) which is a fundamental component of the bacterial cell wall. In turn, RIG-I functions as a sensor of the short (<300 bp) 5′ triphosphorylated dsRNA or ssRNA present within viral RNA. During infection, NLRs and RLRs do not operate separately but as part of the concerted cross-talking program mediated by a variety of PRRs, particularly TLRs. Some NLR family members are not exactly PRRs but are adaptor proteins that facilitate the signaling functions of upstream PRRs. Furthermore, some NLRs are believed to act as transcription factors in the nucleus to promote the expression of MHC genes, and others to activate NF-kB-dependent cytokine expression during infections with any bacteria that enter the cytosol^25^. In light of the above, there is a need to better understand the nature of NLR and RLR expression in different cells, as although these receptors have been identified in various immune cells, knowledge surrounding their activities remains far from sufficient.

The current study demonstrates that *in vivo* differentiated mature tissue MCs isolated from rat peritoneal cavity express NLRs, i.e., NOD1 and NOD2, both at mRNA and protein level. Confocal imaging study indicated that NOD1 is strongly associated with the cytoplasm located near the cell nucleus. Likewise, confocal microscopy analysis showed that NOD2 is predominantly linked with the cytosol and the endoplasmic reticulum.

So far, only a handful of studies have indicated the expression of NLRs in MCs, with Zabucchi *et al*.^26^ being possibly the only study to report that native rat PMCs express NOD1 and NOD2 proteins, which is consistent with our present findings. The authors note that these receptors are associated with the same subcellular compartments, such as the surface and matrix of secretory granules or with cytoplasmic vesicles, and that the free cytosolic NLRs are rare in these locations. NLRs have been confirmed in HMC-1, P815 cells, human cord blood MCs (CBMCs), and MCs from human colon specimens. qPCR examination has identified NOD2 mRNA in HMC-1^27^ and P815 cells^28^, while NOD1^29^ and NOD2 have been confirmed in FACS-sorted human CBMCs^29,30^. An investigation of sequential sections of healthy human colon specimens with anti-tryptase and anti-NOD2 monoclonal antibodies revealed the presence of NOD2 in MCs^31^.

Our findings also indicate that the studied PMCs constitutively express RIG-I mRNA and protein. RIG-I was found to be located not only in the cytosol but also under the cell surface of native cells. These observations are in agreement with those of Mukherjee *et al*.^32^, who found RIG-I to be associated with the actin cytoskeleton in non-polarized epithelial cells, and localizes predominantly to actin-enriched membrane ruffles. Previously, the expression of RIG-I mRNA has only been confirmed in mature human MCs^33^ and human CBMCs^34^; however, Fukuda *et al*.^35^ report that native mouse bone marrow-derived MCs (BMMCs) express RIG-I mRNA and protein.

LL-37 is known to influence TLR levels directly, enhancing TLR2, TLR4 and TLR9 expression on the surface of MCs, and TLR3, TLR5 and TLR7 in their interior^22^. These findings led us to investigate whether LL-37 and another HDP, i.e., defensin hBD-2, exert a similar effect on NLR and RLR expression. The main finding of the present study is that LL-37 and hBD-2 actively modulate the expression and distribution of NOD1, NOD2, and RIG-I in rat PMCs. Flow cytometry and confocal imaging analysis found that LL-37 and hBD-2 up-regulate intracellular NOD1 and NOD2 expression. The results of the microscopy analysis showed that, in addition to increasing the baseline level, hBD-2 could cause translocation of NOD2 from the cytosol to plasma membrane. Also, prolonged LL-37 and hBD-2 treatment was found to enhance the intracellular expression of RIG-I. Because no changes in mRNA were noted we hypothesize that transcription remains unchanged and the pool of already synthesized 0mRNA might be efficiently translated. As a result, protein product accumulation level increase. Another possibility is that mRNA is more preferentially translated than DNA transcribed during the condition we analyzed. Which means that the efficiency of translation is higher than the efficiency of transcription. Therefore, we hypothesize that increased expression of NOD1, NOD, and RIG-I proteins may be the result of the synthesis of new molecules from the pool of mRNA present in the cells. Furthermore, it is hard to find out which component of the transcription/translation pathway the used peptides are working on. Finally, both LL-37 and hBD-2 may inhibit the ubiquitination process which the receptors are undergoing^36^. Which mean an extended half-life of the protein and inhibition of the protein degradation process.

There is little information available on the factors that influence NLR and RLR expression in MCs. Xie *et al*.^28^ found NOD2 mRNA expression to be increased considerably in P815 cells after *Staphylococcus aureus* infection. Okumura *et al*.^31^ stressed that NOD2 mRNA and protein expression was markedly higher in human lung MCs treated with interferon gamma (IFN-γ) than in IFN-γ-untreated MCs. In turn, RIG-I level increased notably upon infection with vesicular stomatitis^35^, Sendai^33^ or denga^34^ virus.

Our present findings indicate that LL-37 and hBD-2 actively stimulate a pro-inflammatory response in rat PMCs: PMC activation *via* LL-37 and hBD-2 resulted in CCL2 and CCL3 release. Additionally, hBD-2, but not LL-37, was found to induce cysLT generation and release. Both peptides, although they belong to the group of antimicrobial peptides, show significant differences within the amino acid sequence. Therefore, their model of action may be slightly different in the case of LT synthesis; LL-37 and hBD-2 may influence other compounds of the pathway leading to LT synthesis. Additionally, so far little is known about the receptor for cathelicidins and defensins on mast cells. There is a high probability that the activation of mast cells under the influence of LL-37 may mediate other membrane molecule/receptor than in the case of hBD-2. PMC activation with LL-37 or hBD-2 also led to dose-dependent and time-dependent PMC degranulation, as revealed by histamine release assessment. A significant new finding was that both peptides were also found to induce ROS generation. These observations support the proposed role of LL-37 and hBD-2 as endogenous amplifiers of the pro-inflammatory response. In addition, it was found that LL-37 and hBD-2 could also stimulate a PMC migratory response, which indicates that these peptides may act as potent chemoattractants, particularly in their milieu.

Although evidence suggests that cathelicidins and defensins can directly activate pro-inflammatory activity in MCs, only a handful of studies so far have investigated MC activation by LL-37 and hBD-2. LL-37 activates LTC_4_ and PGE_2_ synthesis and release in LAD2 cells^37^ and stimulates the production of certain cytokines, such as IL-1β, IL-2, IL-4, IL-5, IL-6, IL-31, TNF, GM-CSF and the chemokine CCL4, in MCs^37–39^. Stimulation with hBD-2, hBD-3, and hBD-4 caused PGD_2_, PGE_2_ and LTC_4_ production in rat MCs^37,40,41^. Niyonsaba *et al*.^37^ observed that hBD-^1–4^ activated IL-2, IL-4, IL-6, IL-8 and IL-31 release in the LAD2 immature human MC line. hBD and LL-37 administration cause MC degranulation, as demonstrated previously by histamine and β-hexosaminidase release assessment^37–43^. It has also been shown that LL-37^44^, hBD-1^45^, hBD-2^45,46^, hBD-3 and hBD-4^40,45^ induce a migratory response in MCs. Also, we previously stated that LL-37 stimulates histamine secretion and TNF and IL-6 release in rat PMCs and increases the mRNA expression of some pro-inflammatory cytokines and chemokines, including IL-1β, CCL2 and CCL3^46^.

In conclusion, our findings demonstrate that *in vivo* differentiated PMCs express NOD1, NOD2, and RIG-I. In addition, the expression of these receptors was found to be enhanced by treatment with the HDPs LL-37 and hBD-2, suggesting that their use may augment the capability of PMCs to detect bacterial and viral PAMPs. Also, as some PRRs are known to recognize endogenous DAMPs, we can hypothesize that some HDPs may drive PMCs to sense cell stress and tissue injury, inducing a non-microbial (sterile) inflammatory response. Our observations show that some HDPs stimulate PMCs to release pro-inflammatory mediators, including ROS, and inducing a migratory response. It should be emphasized that most studies on PRR receptors were performed on MC lines or immature MCs. The cellular models representing MCs, such as immortalized MC lines or MCs cultured and maturated in *in vitro* laboratory conditions, which never accurately reflects physiological conditions, are far away from mast cells found *in vivo* regarding biological properties. Thus, it is believed that MCs isolated from different tissues that developed and differentiated under the influence of microenvironmental factors are the suitable model to study general features of mast cell biology and aspects of mast cell functions within tissues. There are studies conducted on matured human MCs isolated from various tissues for example skin and lungs. However, MC isolation procedure from those tissues is time-consuming, its efficiency is low and isolated MCs have poor viability^47^. That is why rodent (murine or rat) MCs, mainly isolated from the peritoneal cavity, are widely used. The Wistar rat model used is a general multipurpose experimental model having a broad application in MC studies. The protocol of rat PMC isolation is effective and isolated cells are characterized by long-term viability. Also, to obtain a proper density of MCs fewer animals need to be euthanized if the study is conducted on a rat instead of a murine model.

In conclusion, our results can indicate that LL-37 and hBD-2 might strongly influence MC features and activity, strengthening their role in the inflammatory process and combating infection, as well as augmenting their response to stressful stimuli.

## Electronic supplementary material

Supplementary Information 
