# Supplementary material for: The RLR/NLR expression and pro-inflammatory activity of tissue mast cells are regulated by cathelicidin LL-37 and defensin hBD-2
Source: Sci Rep. 2018 Aug 6;8:11750. doi: 10.1038/s41598-018-30289-w (PMC6079022; doi:10.1038/s41598-018-30289-w)

The RLR/NLR expression and pro-inflammatory activity of tissue mast cells are regulated by cathelicidin LL-37 and defensin hBD-2

Justyna Agier,[1] **Sylwia Różalska,**[2] **Magdalena Wiktorska,**[3] **Paulina Żelechowska,**[1] **Joanna Pastwińska,**[1,4] **Ewa Brzezińska-Błaszczyk**[1]

[1]Department of Experimental Immunology, Faculty of Health Sciences, Medical University of Lodz, Lodz, Poland

[2]Department of Industrial Microbiology and Biotechnology, Faculty of Biology and Environmental Protection, University of Lodz, Lodz, Poland

[3]Department of Molecular Cell Mechanisms, Faculty of Health Sciences, Medical University of Lodz, Lodz, Poland

[4]Laboratory of Cellular Immunology, Institute of Medical Biology, Polish Academy of Sciences, Lodz, Poland

Keywords: Mast cells · NOD1 · NOD2 · RIG-I · Defensin · Cathelicidin

Correspondence:

Prof. Ewa Brzezińska-Błaszczyk, PhD

Department of Experimental Immunology, Medical University of Lodz

Pomorska 251, 92-213 Lodz, Poland

Phone: +48 42 272 57 97

e-mail: ewab@csk.umed.lodz.pl

Supplemental Figure S1

Full length blots of Figure 1b. Red dotted lines show the cropping locations.

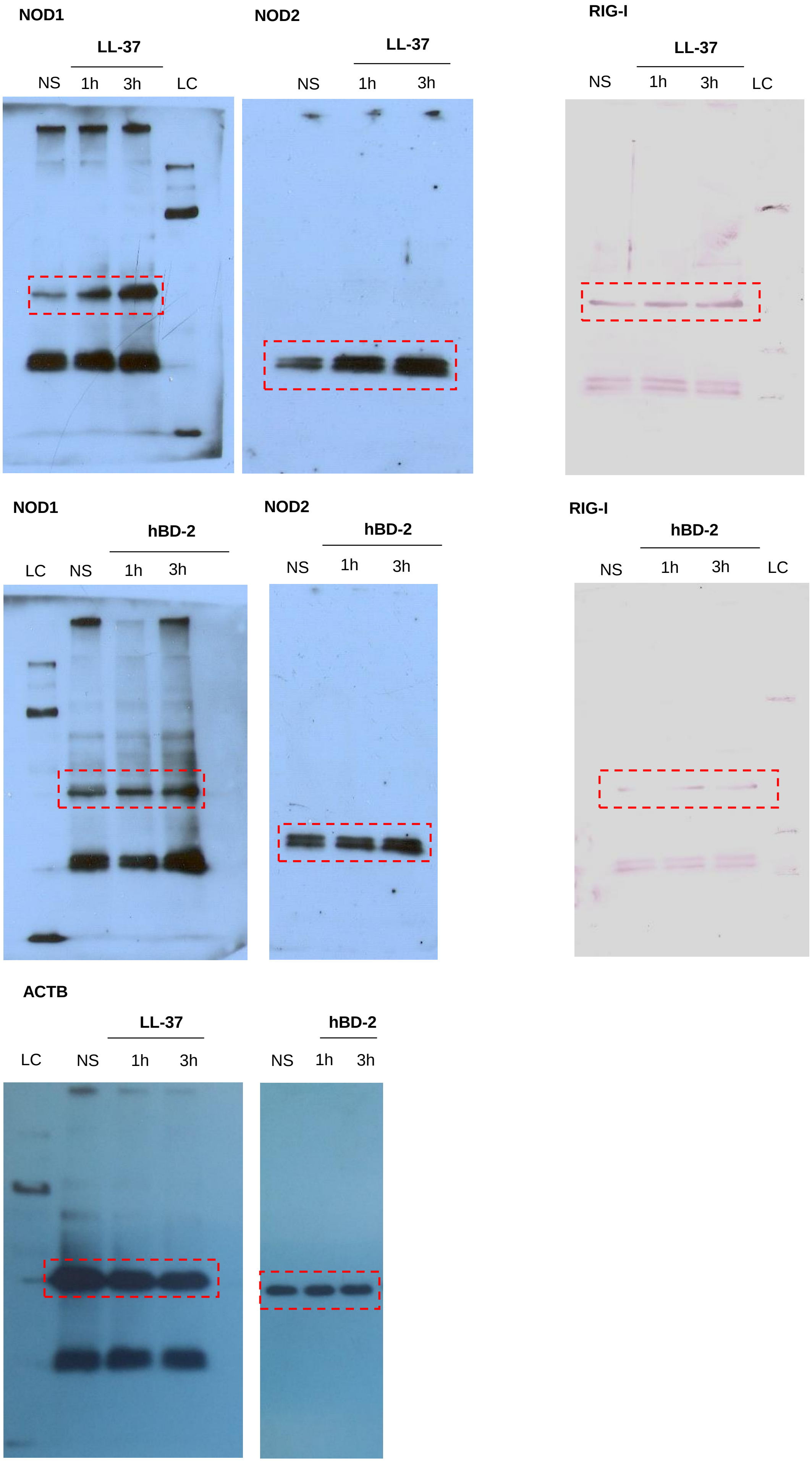

Supplement: Supplementary file 1 — Supplementary Information [file 41598_2018_30289_MOESM1_ESM.pdf]
